# Supplementary material for: Dietary-Induced Ketogenesis: Adults Are Not Children
Source: Nutrients. 2021 Sep 2;13(9):3093. doi: 10.3390/nu13093093 (PMC8467931; doi:10.3390/nu13093093)
Supplement: Supplementary file 1 [file nutrients-13-03093-s001.zip › nutrients-1357718-supplementary.pdf]

Supplementary Material for "Dietary-induced ketogenesis: adults are not children", by *Porper et. al.*

Figure S1 – The relationship between ketone levels in urine and blood amongst pediatric patients. 2

Table S1- Ongoing clinical trials using ketogenic diets in various adult conditions 3

Table S2 - Variations of the Ketogenic diet used in our research: 12

Table SA- Baseline Demographics of adult population: 13

Table SB - Baseline Demographics of children population: 14

Table S4- Dietary intake of the two populations prior to and during diet intervention: 15

Table S5: Anthropometric and metabolic parameters at baseline (week 0) and during treatment (weeks 2-4). 16

Table S6.A- Clinical experience of dietary ketogenesis in healthy adults 17

Table S6.B- Clinical experience of dietary ketogenesis in adults with epilepsy or diabetes 18

Table S6.C-Clinical experience of dietary ketogenesis in adults with cancer 19

Table S6.D- Preclinical studies of dietary/fasting ketogenesis in young vs adult rats 20

References for supplementary material 21

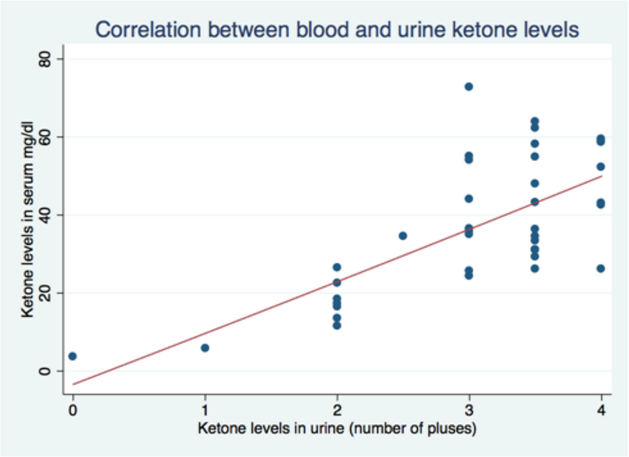

Figure S1 – The relationship between ketone levels in urine and blood amongst pediatric patients.

The scatter plot demonstrates datapoints from the study during which ketone levels were measured concurrently in both venous blood and urine. The data was used to construct a regression line in order to estimate blood ketone levels for those timepoints for which only urine measurements were available.

Table S1- Ongoing clinical trials using ketogenic diets in various adult conditions

NCT Number	Conditions	Interventions
NCT03433261	CNS Oxygen Toxicity, Ketogenic Diet	Dietary Supplement: Ketogenic Diet
NCT03775252	Migraine Disorders	Dietary Supplement: Ketogenic diet
NCT04165707	Overweight and Obesity|Ketogenic Diet	Device: Keyto device + app|Other: Weight Watchers app
NCT00575146	Recurrent Glioblastoma	Dietary Supplement: TAVARLIN
NCT03244735	Chronic Cluster Headache	Other: Atkins diet
NCT01865162	Glioblastoma Multiforme	Other: ketogenic diet
NCT01997749	Acute Stroke	Dietary Supplement: Ketocal 4:1 (Nutricia)|Dietary Supplement: Control diet: Regular diet offered at the hospitals|Dietary Supplement: Ketogenic meals
NCT02763553	Ketosis	Dietary Supplement: Ketogenic Feed|Dietary Supplement: Standard Feed
NCT03285152	Endometrial Cancer	Other: Ketogenic Diet (KD)|Other: Standard Diet (SD)
NCT02983942	Primary Central Nervous System Lymphoma	Dietary Supplement: ketogenic diet|Dietary Supplement: Routine diet
NCT04791787	Glucose Metabolism Disorders (Including Diabetes Mellitus)|Energy Supply; Deficiency|Type 2 Diabetes	Other: Standard weight maintaining diet|Other: Weight maintaining isocaloric ketogenic diet|Dietary Supplement: Beta-hydroxy butyrate
NCT01016522	Amyotrophic Lateral Sclerosis	Dietary Supplement: KetoCal
NCT03255031	Alcoholism	Other: Ketogenic Diet (KD) / Standard American (SA) Snacks and Shakes
NCT03076060	Migraine|Overweight and Obesity	Behavioral: Ketogenic diet|Behavioral: Sham Diet
NCT02516501	Neoplasms	Dietary Supplement: MyAmino|Dietary Supplement: betaquik|Radiation: Radio(chemo)therapy
NCT04530032	Traumatic Brain Injury	Other: ketogenic diet|Other: Standard diet
NCT04615975	Covid19	Dietary Supplement: Ketogenic diet with phytoextracts
NCT01906398	Epilepsy	Other: ketogenic diet

NCT03873922	Psychosis; Acute|Psychosis|Psychotic Disorders|Schizophrenia	Other: Ketogenic diet intervention
NCT02069197	Obesity|Diabetes|Obstructive Sleep Apnea	Other: Ketogenic diet|Drug: Orlistat|Other: Standardized diet
NCT01975766	Head and Neck Neoplasms	Dietary Supplement: Ketogenic diet|Device: External beam radiation therapy
NCT04108819	Obesity Hypoventilation Syndrome|Ketogenic Dieting|Hypercapnic Respiratory Failure	Dietary Supplement: Ketogenic Diet
NCT01796574	Status Epilepticus|Seizure|Epilepsy|Refractory Status Epilepticus|Medically Resistant Status Epilepticus	Dietary Supplement: Ketogenic diet
NCT04316520	Metastatic Renal Cancer	Dietary Supplement: Ketogenic diet
NCT04322110	Liver Fat	Other: PronoKal Method|Other: Low calorie diet
NCT03451799	GBM|Glioblastoma	Other: Ketogenic Diet|Radiation: Standard-of-care radiation|Drug: Standard-of-care Temozolomide
NCT01419483	Pancreatic Neoplasms	Dietary Supplement: Ketogenic diet
NCT02302235	Glioblastoma Multiforme of Brain	Other: Ketogenic Diet|Other: Standardized Diet
NCT03955068	Recurrent Brain Tumor, Childhood|Ketogenic Diet	Other: Strict Classic Ketogenic Diet
NCT03982602	Traumatic Brain Injury|Ketogenic Dieting	Drug: Ketogenic diet
NCT04701957	Alzheimer Disease, Early Onset	Behavioral: Ketogenic diet
NCT03991897	Lymphedema|Ketogenic Dieting	Dietary Supplement: Ketogenic diet|Dietary Supplement: Isocaloric diet
NCT04292938	Glycogen Storage Disease	Dietary Supplement: Low carbohydrate ketogenic diet
NCT02939378	Glioblastoma Multiforme	Dietary Supplement: Ketogenic diet|Dietary Supplement: Standard diet
NCT04235699	Heart Failure With Preserved Ejection Fraction	Other: Nutritional and Dietary Manipulation
NCT03859245	Diabetic Retinopathy (DR)|Age-related Macular Degeneration (AMD)|Mid-peripheral Drusen	Behavioral: Photobiomodulation|Behavioral: Ketogenic diet

	Formation|Diabetic Macular Edema (DME)	
NCT01419587	Carcinoma, Non-Small-Cell Lung	Dietary Supplement: Ketogenic diet
NCT01754350	Recurrent Glioblastoma	Dietary Supplement: calorie-restricted ketogenic diet and transient fasting|Dietary Supplement: standard nutrition
NCT01364545	Parkinson's Disease	Dietary Supplement: Ketone ester drink|Dietary Supplement: Placebo (carbohydrate containing) drink
NCT03843606	McArdle Disease	Other: Modified ketogenic diets
NCT04044508	McArdle Disease	Dietary Supplement: Ketocal 4:1 liquid Nutricia (intervention)|Dietary Supplement: Fortini multifibre Nutrica (placebo)
NCT03535701	Stage IV Breast Cancer AJCC v6 and v7	Dietary Supplement: Dietary Intervention|Other: Laboratory Biomarker Analysis|Drug: Paclitaxel|Other: Quality-of-Life Assessment|Other: Questionnaire Administration
NCT02046187	Glioblastoma (GBM)	Dietary Supplement: Ketogenic Diet|Radiation: Radiation therapy|Drug: Temozolomide
NCT04231734	Mantle Cell Lymphoma|Ketogenic Dieting	Other: Ketogenic Diet
NCT03901014	Cholesterol Metabolism|Ketone Metabolism	Other: Standard American diet|Other: Very low carbohydrate diet
NCT04714541	Anorexia Nervosa	Drug: Ketamine Hcl 50Mg/Ml Inj
NCT03691558	Ketogenic Dieting|Physical Activity	Other: Ketogenic diet|Other: Western Diet
NCT04750941	Follicular Lymphoma|Endometrial Cancer	Drug: Copanlisib|Other: Ketogenic Diet
NCT04382183	Obesity	Behavioral: Ketogenic weight loss maintenance diet|Behavioral: Isocaloric balanced weight loss maintenance diet
NCT03791242	Obstructed Sleep Apnea Syndrome in Patient Candidate to Baratric Surgery	Device: Cpap and ketogenic diet
NCT04691960	Glioblastoma	Other: Ketogenic Diet|Drug: Metformin
NCT04629365	Diet, Healthy|Body Weight	Other: Ketogenic Diet|Other: Normal Diet
NCT04396015	Cognitive Function 1, Social	Dietary Supplement: Bulletproof Brain Octane|Dietary Supplement: Placebo

NCT04383951	NASH - Nonalcoholic Steatohepatitis|Cirrhosis, Liver	Other: Dietary consult for participants in the ketogenic diet arm|Other: Dietary consult for participants in the standard of care arm
NCT04801173	PCOS|Obesity|Insulin Resistance	Combination Product: Very low calorie ketogenic diet|Behavioral: Low calorie standard diet
NCT03962647	Estrogen Receptor-positive Breast Cancer	Dietary Supplement: 2-Week Ketogenic Diet|Drug: Letrozole
NCT03278249	Glioblastoma	Other: Modified Atkins Ketogenic Diet
NCT04019431	Obesity	Dietary Supplement: Whey protein, vegetable protein or animal protein
NCT03935854	Obesity|Ketogenic Dieting|Metabolic Syndrome|Bipolar Disorder|Schizophrenia|Weight Gain|Psychotropic Drug, Unspecified Causing Adverse Effects in Therapeutic Use	Other: LCHF, Ketogenic Diet
NCT00498394	Obesity	Behavioral: Clinical diet trial
NCT03338452	Obesity	Behavioral: Low energy ketogenic diet
NCT00108524	Diabetes Mellitus|Obesity	Behavioral: Low carbohydrate ketogenic diet|Drug: Orlistat|Behavioral: Low-fat diet
NCT00188500	Epilepsy	Behavioral: atkins diet|Behavioral: ability to follow low carbohydrate diet
NCT04078971	Athletic Performance	Other: Western diet|Other: ketogenic diet
NCT03690193	Alzheimer Disease	Behavioral: Ketogenic Diet
NCT03509571	Spinal Cord Injuries	Other: Ketogenic Diet|Other: Standard Diet
NCT04163120	Polycystic Ovary Syndrome	Other: Low calorie Mediterranean ketogenic diet with phytoextracts (KEMEPHY)
NCT04705558	PreDiabetes	Other: continuous moderate intensity aerobic training|Other: ketogenic diet
NCT03160599	Malignant Tumors	Other: ketogenic diet
NCT04019730	Upper Respiratory Tract Infections	Other: diet
NCT02417350	Healthy	Other: Ketogenic diet for four weeks, then a 15-week washout, and finally a NFA recommended diet for four weeks|Other: NFA recommended diet for four weeks,

		then a 15 week washout, and finally a ketogenic diet for four weeks.
NCT03075514	Glioblastoma|Glioblastoma Multiforme|Glioblastoma, Adult	Other: MKD|Other: MCT
NCT03196271	Intractable Epilepsy	Dietary Supplement: Ketocal 2.5:1
NCT03328858	Brain Tumors	Other: Ketogenic Diet
NCT01716468	Cancer	Other: Ketogenic Diet
NCT02497105	Epilepsy	Other: Ketogenic Diet
NCT02477904	Autism Spectrum Disorder	Other: Ketogenic Diet
NCT03784716	Fatty Liver, Nonalcoholic|Liver Diseases	Other: Ketogenic Diet|Other: Standard Weight Loss Diet
NCT03031028	Refractory Epilepsy	Other: ketogenic diet
NCT04175964	Polycystic Ovary Syndrome	Behavioral: ketogenic diet|Combination Product: caloric diet with Metformin|Behavioral: caloric diet
NCT04631445	Metastatic Pancreatic Ductal Adenocarcinoma	Other: Ketogenic Diet
NCT02644239	Epilepsy|Cardiovascular Disease|Non-alcoholic Fatty Liver Disease|Quality of Life	Dietary Supplement: ketogenic diet
NCT02835820	HIV|Neurocognitive Impairment	Dietary Supplement: Ketogenic Diet
NCT03301532	GLUT1DS1	Drug: Triheptanoin
NCT03652649	Type2 Diabetes Mellitus|Obesity	Other: 3:1 ratio Ketogenic diet
NCT04086498	Overweight|Mood|Appetitive Behavior	Other: Mediterranean diet|Other: KEMEPHY|Other: KD
NCT04492228	Covid19|Ketogenic Dieting	Other: Ketogenic diet
NCT01538355	Relapsing-Remitting Multiple Sclerosis	Other: Prolonged Fasting|Other: Ketogenic low glycemic load treatment|Other: Control diet
NCT03114176	Overweight and Obesity|Exertion; Excess|Oxygen Toxicity	Dietary Supplement: Ketogenic Diet
NCT00476125	Metabolic Regulation|Fasting|Ketogenic Diet	Behavioral: Dietary manipulation ketogenic diet|Behavioral: Dietary Manipulation - 12 day ketogenic diet|Behavioral: 16 Hour Fast
NCT01092247	Malignant Tumors	Other: Nutritional support with Standard diet|Other: Nutritional intervention with the Ketogenic diet

NCT02744079	Breast Neoplasms	Behavioral: Low carbohydrate diet|Behavioral: Low fat diet
NCT04083352	PTSD|Ketosis	Dietary Supplement: Pruvit Ketomax Ketone Salt|Dietary Supplement: Placebo
NCT04308577	Brain Injuries|Traumatic Brain Injury|Subarachnoid Hemorrhage|Stroke|Anoxic Brain Injury|Neuroinfections	Other: Ketogenic diet with added MCT
NCT03530501	Weight Loss|Inflammation	Other: Diet|Dietary Supplement: Synbiotic1|Dietary Supplement: Synbiotic2
NCT00415688	Type 2 Diabetes Mellitus|Overweight|Obesity	Behavioral: Low carbohydrate, ketogenic diet|Behavioral: Low glycemic index, reduced calorie diet
NCT01235208	Overweight|Obesity|Metabolic Syndrome	Other: The "Dr. Fedon Lindbergs Vektcoach" Treatment
NCT04646733	Weight Loss|Body Composition	Other: High Protein Diet Group|Other: No High Protein Diet Group
NCT03202108	Ketogenic Dieting|Epilepsy|Glucose Transporter Type 1 Deficiency Syndrome	Dietary Supplement: Krio
NCT02915211	Intractable Epilepsy|Glucose Transporter Type 1 Deficiency Syndrome|Ketogenic Dieting	Dietary Supplement: Keyo
NCT03014752	Epilepsy Intractable	Other: Classical ketogenic diet|Other: Modified Atkins diet
NCT04399954	Epilepsy Intractable|Glucose Transporter Type 1 Deficiency Syndrome|Ketogenic Dieting	Dietary Supplement: Ketoflo
NCT01983163	Epilepsy	Other: Ketogenic diet (2.5 to 4:1)|Other: Modified Atkin's diet
NCT02092753	Quality of Life	Other: Standard diet (SD)|Other: Experimental 1: Ketogenic diet (KD).|Other: Experimental 2: "Low glyc[illegible]mic and insulinemic" diet (LOGI)
NCT04461938	Glioma, Mixed	Other: Fasting
NCT03785808	Diet Modification|Lung Cancer	Behavioral: Special Diet Therapy (low-carb)|Behavioral: Special Diet Therapy (low-fat)|Behavioral: Special Diet Therapy (USDA control)
NCT03171506	Ovarian Cancer|Endometrial Cancer	Other: Ketogenic diet|Other: AND diet

NCT01535911	Glioblastoma	Other: Energy restricted Ketogenic Diet (ERKD) (Metabolic Nutritional Therapy)
NCT04309149	Ketosis|Epilepsy|Ketogenic Diet|Fatty Acid Oxidation Disorder|Malabsorption	Dietary Supplement: MCT fats
NCT02216500	Seizures	Dietary Supplement: Ketogenic Therapy
NCT03810378	Diabetes Mellitus, Type 2|PreDiabetes	Behavioral: Mediterranean Diet (Med-Plus)|Behavioral: Well-Formulated Ketogenic Diet (WFKD)
NCT04309214	Ketosis|Epilepsy|Fatty Acid Oxidation Disorder|Malabsorption|Ketogenic Diet|Medium Chain Triglycerides	Dietary Supplement: MCT fats
NCT04195594	Ketogenic Dieting|Healthy	Other: Nic's Keto Diet
NCT02286167	Glioblastoma Multiforme	Other: Diet modification
NCT04705298	Drug Resistant Epilepsy	Dietary Supplement: Prebiotic|Dietary Supplement: Placebo
NCT04584346	Parkinson's Disease	Dietary Supplement: Liquigen MCT oil
NCT02409784	Healthy	Dietary Supplement: Pre-KD|Dietary Supplement: Post-KD
NCT04744558	Ketogenic Dieting	Other: Low carb high fat ketogenic dieting|Other: Low carb high fat non-ketogenic dieting
NCT04943926	Diabetes Mellitus, Type 2|Overweight and Obesity|Hypertension|Glucose Intolerance|Insulin Resistance|Dyslipidemia Associated With Type II Diabetes Mellitus	Other: Energy restricted diet|Other: Low carbohydrate high fat diet
NCT01820663	Stroke|Ischemic Stroke	Other: Modified Atkins Diet|Other: Control diet
NCT01278966	Epilepsy	Other: The Modified Atkins Diet
NCT03665948	Nutrition|Ketogenic Dieting|Low Glycemic Index Diet|Sport|Crossfit	Other: Dietary Intervention - Ketogenic Diet (KD)|Other: Dietary Intervention - Low-Glycemic Index Diet (CHO-LGI)
NCT04730869	Glioblastoma Multiforme	Other: Standard Treatment Plus Metabolic Therapy Program
NCT04680780	ADPKD	Other: Ketogenic diet|Other: 3-days water-fasting|Other: Control
NCT03394664	Obesity	Behavioral: Feeding Study

NCT04260542	Insulin Resistance|Obesity	Behavioral: Fasting/refeeding|Behavioral: Ketogenic diet/ fasting
NCT04472624	ADPKD	Other: Fasting|Other: Ketogenic diet
NCT01311440	Epilepsy|Focal Epilepsy|Generalized Epilepsy	Other: Modified Atkins diet treatment
NCT04358835	COVID-19	Dietary Supplement: Ketogenic diet|Other: standard of care
NCT03679260	Prostate Cancer	Other: Carbohydrate restricted diet|Other: Non-restricted diet|Other: Phone counseling with dietitian
NCT02426047	Epilepsy|Seizure|Catamenial Epilepsy|Medically Resistant Epilepsy|Medically Resistant Seizures	Dietary Supplement: betaquik®
NCT04920058	Metabolic Syndrome	Device: Continuous Glucose Monitor|Other: <Active Comparator?>
NCT03319173	Mild Cognitive Impairment|Metabolic Syndrome	Behavioral: Dietary intervention
NCT02944253	Obesity	Other: Low energy diet 70 gram carbohydrates|Other: Low energy diet 100 gram carbohydrates|Other: Low energy diet 130 gram carbohydrates
NCT03934476	Obesity|Diet Modification	Behavioral: Ketogenic diet|Behavioral: Exercise HIIT
NCT03998878	Obesity	Behavioral: Low-Carbohydrate Diet|Behavioral: Intermittent Energy Restriction|Behavioral: Hunger Training
NCT03710928	Type1diabetes	Other: very low carbohydrate diet|Other: standard carbohydrate diet
NCT04200391	Type1diabetes	Other: Very low carbohydrate diet
NCT04219709	Type1diabetes	Other: Very low carbohydrate diet|Other: Standard carbohydrate diet
NCT04678713	Obesity	Dietary Supplement: investigate the nutritional determinants of MFO in moderate to well-train individuals|Other: Investigate the physiological determinants of MFO in moderate to well-train individuals
NCT03231514	Ketogenic Dieting	Dietary Supplement: Carbohydrate Supplement (Carb10)|Other: Diet|Other: Exercise

NCT02984540	Mild Cognitive Impairment|Insulin Resistance	Other: Low-Carbohydrate Diet|Other: Low-Fat Diet
NCT02825758	Deficiency of Micronutrients	Dietary Supplement: ZestiVits
NCT03878225	Alcohol Use Disorder|Alcohol Withdrawal|Ketosis	Dietary Supplement: H.V.M.N. Ketone Ester|Other: Placebo

Table S2 - Variations of the Ketogenic diet used in our research:

Diet *	Composition (%)**	Principles	Meal example
Classic ketogenic diet<br><br>Wilder, 1921<br><br>[1,2]	90% fat,<br>8% protein,<br>2% carbohydrate.	A strict ratio, based on mass, between fat to both protein and carbohydrates (3: 1, 4: 1 respectively.<br><br>Requires hospitalization to initiate the diet, and weighing individual food items.	30 g egg,<br>15 g cheese 30% fat,<br>20 g heavy cream 38% fat,<br>9 ml olive oil,<br>40 g tomato.
Modified Atkins diet<br><br>Kossoff, 2003<br><br>[3-5]	64% fat,<br>30% protein,<br>6% carbohydrate.	Limit carbohydrates to 10-15 grams a day.<br><br>No caloric and protein restriction.<br><br>Neither hospitalization, nor weighing individual food items required.	1/4 cup heavy cream 38% fat,<br>omelette with butter,<br>slice of yellow cheese 28% fat,<br>four tablespoons of cream cheese 30% fat.

*Medium chain triglyceride (MCT) coconut-extracted oil thought to stimulate hepatic production of β-hydroxybutyrate (β-OHB) ketone bodies [6,7] was added at the dietician's discretion.

**Data refer to % contribution to total caloric intake

Table S3.A- Baseline Demographics of adult population:

patient #	Age	Sex	BMI	Weight (kg)	Original Histology	Concomitant therapy	Metabolic intervention therapy
1	62	m	28.7	79	GBM	Bev + steroids	**Cohort 1:** MAD alone
2	61	m	24.9	75.7	GBM	Bev	
3	66	f	32.6	78.4	GBM	-	
4	61	f	31.6	81	AA	-	**Cohort 2:** MAD + Metformin 850mg twice-daily
5	58	m	26.6	88	GBM	Rindopepimut	
6	57	f	26	70	AA	Steroids	
7	58	m	31.9	103.4	GBM	Bev	
8	62	m	31.7	97	GBM	Tmz	
9	58	m	28.9	83.6	GBM	Tmz	
10	74	f	29.9	70	GBM	Tmz + steroids	
11	52	f	24.1	67.9	AA	Tmz	**Cohort 3:** MAD + Metformin 850 mg three-times daily
12	66	m	24.3	77.9	GBM	Tmz	
13	63	m	38.6	120	GBM	Tmz +steroids	

Abbreviations: AA- Anaplastic Astrocytoma Grade 3, GBM- Glioblastoma, Bev bevacizumab, Tmz- Temozolomide, MAD-modified Atkins diet.

*For additional therapeutic details see [8]

Table S3.B - Baseline Demographics of children population:

patient #	Age	Sex	Height (cm)	Weight (kg)	Etiology	Number of antiepileptic treatments	Diet type
1	4	f	-	12.75	Rett syndrome	3	CKD
2	4.5	f	103	19.5	CMV	3	CKD
3	1.7	m	98.5	9.2	GPI Deficiency	3	CKD
4	11.4	m	136	30.3	Unknown	2	CKD/MAD
5	3.8	m	92.5	13.7	Unknown	0	CKD/MAD
6	1.8	f	78	8.4	PDHA	4	CKD
7	0.8	f	72	8	SCN2A mutation	0	CKD
8	2.7	f	80	10	GLUT1DS	1	CKD/MAD
9	3.8	m	-	15.5	Unknown	1	CKD
10	1.4	f	-	7.2	Asphyxia	3	CKD
11	1.0	m	-	9.4	Asphyxia	3	CKD
12	0.4	m	-	-	KCNQ2 mutation	2	CKD
13	8.5	f	-	-	Unknown	3	MAD
14	2.3	f	--	11.4	Chronic encephalitis	3	CKD
15	2.0	m	83	11.1	KCNQ2 mutation	4	CKD

Abbreviations: CMV-Cytomegalovirus, GPI -Glucose-6-phosphate isomerase, GLUT1DS- GLUT1 deficiency syndrome, PDHD- pyruvate de hydrogenase deficiency, CKD -classical ketogenic diet, MAD-modified Atkins diet.

Table S4- Dietary intake of the two populations prior to and during diet intervention:

Population		Prior to diet (%)*	After 1 month on diet (%)	p value
**Children**	Carbohydrates	51.7 ± 8.2	4.1 ± 2.0	<0.00001
	Fats	35.8 ± 6.5	88.7 ± 2.3	<0.00001
**Adults**	Carbohydrates	30.5 ± 3.7	5.7±0.5	0.0001
	Fats	49.2±15.5	73.8±9.4	0.0025

*Data refer to % contribution to total caloric intake

Table S5: Anthropometric and metabolic parameters at baseline (week 0) and during treatment (weeks 2-4).

population	Metabolite	Units	Mean - Baseline	Mean- During treatment	p value
**Adults**	Weight	kg	85.1	84.4	0.4
	BMI	kg/m^2	29.48	29.16	0.3
	Glucose	mg/dl	99.8	96.2	0.4
	HbA1C	%	5.6	5.4	0.2
	Insulin	mU/l	14	11.4	0.2
	C peptide	µg/l	2.97	2.76	0.5
	β-OHB	mmol/l	0.09	0.59	0.006
**Children**	Weight	kg	10.8	11.2	0.014
	β-OHB	mmol/l	0.15	3.4	<0.0001

Abbreviations: BMI Body Mass Index, HbA1C Hemoglobin A1C, β-OHB β-hydroxybutyrate.

Additional details of anthropometric and metabolic parameters in the adult population are provided in reference [8].

Table S6.A- Clinical experience of dietary ketogenesis in healthy adults

Type of diet inducing ketosis	Population studied	n	Achieved ketone levels	Effect of age	Additional Outcomes	Reference
Hyper triglyceridemic clamp (primed infusion of a long-chain triacylglycerol emulsion	Ten healthy elderly men (70–78 y) and 10 healthy young men (19–45 y)	20	Plasma β-OHB levels were at the range of 0.6-0.8 Mm/L, versus 0.4-0.6 m/L during infusion	In the basal state Plasma β-OHB levels were higher in the elderly than in the young subjects and were increased in a similar manner in both groups during infusion	Plasma lipoprotein lipase activity was almost three times higher in the young than in the elderly subjects ($P < 0.001$)	[9]
Ketogenic meal (4.5:1 ratio), 90% fat containing MCT	Healthy subjects in three age groups (76 y, n=11 vs 50 y n=12, or 23 y n=9)	32	Plasma β-OHB levels were at the range of 0.5-1.5 Mm/l	There was no difference in Plasma β-OHB levels during the 6 h after KD meal between the three groups	There was no difference in cumulative 13C- β-OHB oxidation between the three groups	[10]
After 6 hours fasting	Twenty healthy young adults (mean age, 26 years) and 24 healthy older adults (mean age, 74 years)	44	Plasma β-OHB levels were 0.32 ± 0.26 mM in the young group versus 0.21 ± 0.1 mM in the adult group	The difference in Plasma β-OHB levels between the two groups was not examined in this study	Rate constants of AcAc (Ka) in the brain were significantly lower in older adults compared with younger adults	[11]
Flaxseed oil supplement	Ten healthy young (25 ± 0.9 years) and 10 older adults (73.1 ± 2.2 years)	20	Plasma β-OHB levels were 1.27 ± 55 mM versus 128 ± 30 after consuming flaxseed oil supplement	Postprandial production of β-OHB was increased by 26% ($P = 0.037$) only in the young adult group	The ratio of plasma fasting b-HB or AcAc to NEFA was higher in the young adult group	[12]

Abbreviations: MCT; medium chain triglyceride, β-OHB; β-hydroxybutyrate, KD; ketogenic diet. NEFA; Non-esterified Free Fatty Acid.

Table S6.B- Clinical experience of dietary ketogenesis in adults with epilepsy or diabetes

Type of diet inducing ketosis	Population studied	n	Achieved ketone levels	Additional Outcomes	Reference
Low carbohydrate diet (less than 30 grams per day) to achieve nutritional ketosis	Patients with a mean age of 53.75 with type 2 diabetes	262	Plasma β-OHB levels were increased from baseline (0.17 ± 0.01 mmol L to 0.54 ± 0.04 mmol L) within 70 days, followed by a decrease at 1 year (0.30 ± 0.02 mmol L), amongst 83% of participants.	Significantly reduce in HbA1c, medication use, and weight within 70 days, and through 1 year	[13]
Classic KD via gastrostomy tube	Patients 18 to 80 years of age with SRSE	15	All patients reached ketosis (defined as urine acetoacetate ≥40 mg/dL and/or serum β-OHB ≥2 mmol/L) in a median of 2 days after KD initiation.<br><br>Not mentioned specific levels	SRSE stopped in 73% of patients, within 1 week of KD initiation.	[14]

Abbreviations: SRSE; super refractory status epilepticus β-OHB; β-hydroxybutyrate, KD; ketogenic diet, HbA1c; Hemoglobin A1c.

Table S6.C-Clinical experience of dietary ketogenesis in adults with cancer

**Type of diet inducing ketosis**	**Population studied**	**n**	**Achieved ketone levels**	**Additional Outcomes**	**Reference**
MAD (20-40g of CHO a day, Unlimited calories) for 16 weeks.	Advanced cancer patients (42–87 years) who were not on chemotherapy (1 with GBM grad 4)	17	Seven out of 10 patients (70 %) achieved a ketone levels of 0.28–0.96mM, with 4 patients (36 %) achieving values between 0.96 to 4.13 mM and only 2 were non-ketotic	Clinical response did not correlate with ketosis or glycemia	[15]
The CHO intake was targeted at no higher than 5% of total energy	Advanced cancers, mixed patients (52-73 years)	10	Blood ketone levels ranged between 0.5-1.5 Mm	The extent of ketosis correlated with stable disease however, did not correlated with changes in weight loss, percentage of calorie deficit, CHO intake, total energy intake, CHO (kcal)/total energy intake, or CHO (kcal)/predicted energy requirements.	[16]
Less than 70 g CHO per day	Advanced cancers, mixed patients (30-65 years)	16	Ketone levels were not measured in blood.<br>Ketosis in urine was achieved in 3 of patients at range between 1.5-4 Mm/l.		[17]
A low-carbohydrate, ketogenic diet containing plant oils	Recurrent glioma patients (30-72 years)	19	Ketone levels were not measured in blood.<br>ketosis in urine was achieved in 12/13 of patients at range between 0.3-1.0 mM/l		[18]

Abbreviations: MAD; modified Atkins diet, GBM; glioblastoma, CHO; carbohydrate

Table S6.D- Preclinical studies of dietary/fasting ketogenesis in young vs adult rats

Animal Model	Age	Method of inducing ketosis	Achieved Ketone levels	Effect of age	Additional outcomes	Reference
Wistar rats with seizures	Adults and young rats	Fed either a 4:1 ratio ketogenic diet (KD) or a 6.3:1 ratio KD	β-OHB levels ranged between 0.5-1.0 mM in the young group versus 3.0-5.0 mM in the older group	In adult rats, not like in young rats, β-OHB levels did not increase into the 2- 4-mM range, perceived to be the "therapeutic range" humans	The 4:1 KD—which did not elevate threshold doses—was able to elevate β-OHB levels even higher than the 6.3:1 KD	[19]
Normal vs streptozotocin diabetic rats	Adult rats (50-week-old) vs young rats (8-week-old)	A 36h fasting period	β-OHB levels were 1.29 ± 15 mM and 3.44 ± 98 mM (fed vs fasting state respectively) in the young group versus 1.10 ± 24 mM and 3.03 ± 55 mM in the older group	Fasting AcAc levels in adult rats were significantly lower than that in young rats; no significant difference observed in β-OHB levels	Total plasma total carnitine tended to decrease with aging. the plasma levels of FFA and glucagon in fasted adult rats were significantly higher than those in young rat.	[20]
Sprague–Dawley rats with seizures	Adults and young rats of the ages 22-126 days post-natal	Fed a calorie-restricted ketogenic diet (more than 78% of its calories from fat)	The mean Ketone levels were 7 mM in the 22 days group versus 0.5 mM in the 126 days group	The blood level of β-OHB in the younger rats was significantly higher than that of the older rates	Ketogenic animals that began the diet at 126 days exhibited no significant elevation in β-OHB compared to controls (normal diet)	[21]

Abbreviations: ACAC; Acetylacetone, β-OHB; β-hydroxybutyrate, FFA; free fatty acid, KD; ketogenic diet.

References for supplementary material

.1 Zupec-Kania, B.A.; Spellman, E. An overview of the ketogenic diet for pediatric epilepsy. *Nutr Clin Pract* **2008**, *23*, 589-596.

.2 Wilder, R. In *The effects of ketonemia on the course of epilepsy*, Mayo Clin Proc, 1921; pp 307-308.

.3 Kossoff, E.H.; Dorward, J.L. The modified atkins diet. *Epilepsia* **2008**, *49 Suppl 8*, 37-41.

.4 Sharma, S.; Sankhyan, N.; Gulati, S.; Agarwala, A. Use of the modified atkins diet for treatment of refractory childhood epilepsy: A randomized controlled trial. *Epilepsia* **2013**, *54*, 4.81-486

.5 Kossoff, E.H.; Cervenka, M.C.; Henry, B.J.; Haney, C.A.; Turner, Z. A decade of the modified atkins diet (2003-2013): Results, insights, and future directions. *Epilepsy Behav* **2013**, *29*, 437-442.

.6 Liu, Y.-M.; Wang, H.-S. Medium-chain triglyceride ketogenic diet, an effective treatment for drug-resistant epilepsy and a comparison with other ketogenic diets. *Biomed J* **2013**, *36*, 9-15.

.7 Seaton, T.B.; Welle, S.L.; Warenko, M.K.; Campbell, R.G. Thermic effect of medium-chain and long-chain triglycerides in man. *The American Journal of Clinical Nutrition* **1986**, *44*, 630-634.

.8 Porper, K.; Shpatz, Y.; Plotkin, L.; Pechthold, R.G.; Talianski, A.; Champ, C.E.; Furman, O.; Shimoni-Sebag, A.; Symon, Z.; Amit, U.*, et al.* A phase i clinical trial of dose-escalated metabolic therapy combined with concomitant radiation therapy in high-grade glioma. *J Neurooncol* **2021**, *153*, 487-496.

.9 Åberg, W.; Thörne, A.; Olivecrona, T.; Nordenström, J. Fat oxidation and plasma removal capacity of an intravenous fat emulsion in elderly and young men. *Nutrition* **2006**, *22*, 738-743.

.10 Freemantle, E.; Vandal, M.; Tremblay-Mercier, J.; Plourde, M.; Poirier, J.; Cunnane, S. Metabolic response to a ketogenic breakfast in the healthy elderly. *JNHA-The Journal of Nutrition, Health and Aging* **2009**, *13*, 293-298.

.11 Nugent, S.; Tremblay, S.; Chen, K.W.; Ayutyanont, N.; Roontiva, A.; Castellano, C.-A.; Fortier, M.; Roy, M.; Courchesne-Loyer, A.; Bocti, C. Brain glucose and acetoacetate metabolism: A comparison of young and older adults .*Neurobiology of aging* **2014**, *35*, 1386-1395.

.12 Hennebelle, M.; Courchesne-Loyer, A.; St-Pierre, V.; Vandenberghe, C.; Castellano, C.-A.; Fortier, M.; Tessier, D.; Cunnane, S.C. Preliminary evaluation of a differential effect of an α-linolenate-rich supplement on ketogenesis and plasma ω-3 fatty acids in young and older adults. *Nutrition* **2016**, *32*, 1211-1216.

.13 Hallberg, S.J.; McKenzie, A.L.; Williams, P.T.; Bhanpuri, N.H.; Peters, A.L.; Campbell, W.W.; Hazbun, T.L.; Volk, B.M.; McCarter, J.P.; Phinney, S.D. Effectiveness and safety of a novel care model for the management of type 2 diabetes at 1 year: An open-label, non-randomized, controlled study. *Diabetes Therapy* **2018**, *9*, 583-612.

.14 Cervenka, M.C.; Hocker, S.; Koenig, M.; Bar, B.; Henry-Barron, B ;.Kossoff, E.H.; Hartman, A.L.; Probasco, J.C.; Benavides, D.R.; Venkatesan, A. Phase i/ii multicenter ketogenic diet study for adult superrefractory status epilepticus. *Neurology* **2017**, 10.1212/WNL. 0000000000003690.

.15 Tan-Shalaby, J.L.; Carrick, J.; Edinger, K.; Genovese, D.; Liman, A.D.; Passero, V.A.; Shah, R.B. Modified atkins diet in advanced malignancies-final results of a safety and feasibility trial within the veterans affairs pittsburgh healthcare system. *Nutrition & metabolism* **2016**, *13*, 52.

.16 Fine, E.J.; Segal-Isaacson, C.; Feinman, R.D.; Herszkopf, S.; Romano, M.C.; Tomuta, N.; Bontempo, A.F.; Negassa, A.; Sparano, J.A. Targeting insulin inhibition as a

metabolic therapy in advanced cancer: A pilot safety and feasibility dietary trial in 10 patients. *Nutrition* **2012**, *28*, 1028-1035.
.17 Schmidt, M.; Pfetzer, N.; Schwab, M.; Strauss, I.; Kämmerer, U. Effects of a ketogenic diet on the quality of life in 16 patients with advanced cancer: A pilot trial. *Nutrition & metabolism* **2011**, *8*, 54.
.18 Rieger, J.; Bähr, O.; Maurer, G.D.; Hattingen, E.; Franz, K.; Brucker, D.; Walenta, S.; Kämmerer, U.; Coy, J.F.; Weller, M. Ergo: A pilot study of ketogenic diet in recurrent glioblastoma. Erratum in/ijo/45/6/2605. *International journal of oncology* **2014**, *44* , .1843-1852
.19 Nylen, K.; Likhodii, S.; Abdelmalik, P.A.; Clarke, J.; Burnham, W.M. A comparison of the ability of a 4:1 ketogenic diet and a 6.3:1 ketogenic diet to elevate seizure thresholds in adult and young rats. *Epilepsia* **2005**, *46*, 1198-1204.
.20 Okuda, Y.; Kawai, K.; Yamashita, K. Age-related change in ketone body metabolism: Diminished glucagon effect on ketogenesis in adult rats*. *Endocrinology* **1987**, *120*, 2152-2157.
.21 Bough, K.J.; Valiyil, R.; Han, F.T.; Eagles, D.A. Seizure resistance is dependent upon age and calorie restriction in rats fed a ketogenic diet. *Epilepsy research* **1999**, *35*, 21-28.
